# Illumina MiSeq sequencing analysis of fungal diversity in stored dates

**DOI:** 10.1186/s12866-017-0985-7

**Published:** 2017-03-27

**Authors:** Ismail M. Al-Bulushi, Muna S. Bani-Uraba, Nejib S. Guizani, Mohammed K. Al-Khusaibi, Abdullah M. Al-Sadi

**Affiliations:** 1 Department of Food Science and Nutrition, College of Agricultural and Marine Sciences, Sultan Qaboos University, P.O. Box-34, Al-Khod, 123 Oman; 2 Department of Crop Sciences, College of Agricultural and Marine Sciences, Sultan Qaboos University, P.O. Box-34, Al-Khod, 123 Oman

**Keywords:** *Phoenix dactylifera* L, Population structure, Fungal diversity, Fungal pathogens, Date palm

## Abstract

**Background:**

Date palm has been a major fruit tree in the Middle East over thousands of years, especially in the Arabian Peninsula. Dates are consumed fresh (*Rutab*) or after partial drying and storage (*Tamar*) during off-season. The aim of the study was to provide in-depth analysis of fungal communities associated with the skin (outer part) and mesocarp (inner fleshy part) of stored dates (*Tamar*) of two cultivars (*Khenizi* and *Burny*) through the use of Illumina MiSeq sequencing.

**Results:**

The study revealed the dominance of *Ascomycota* (94%) in both cultivars, followed by *Chytridiomycota* (4%) and *Zygomycota* (2%). Among the classes recovered, *Eurotiomycetes*, *Dothideomycetes, Saccharomycetes* and *Sordariomycetes* were the most dominant. A total of 54 fungal species were detected, with species belonging to *Penicillium, Alternaria, Cladosporium* and *Aspergillus* comprising more than 60% of the fungal reads. Some potentially mycotoxin-producing fungi were detected in stored dates, including *Aspergillus flavus, A. versicolor* and *Penicillium citrinum,* but their relative abundance was very limited (<0.5%). PerMANOVA analysis revealed the presence of insignificant differences in fungal communities between date parts or date cultivars, indicating that fungal species associated with the skin may also be detected in the mesocarp. It also indicates the possible contamination of dates from different cultivars with similar fungal species, even though if they are obtained from different areas.

**Conclusion:**

The analysis shows the presence of different fungal species in dates. This appears to be the first study to report 25 new fungal species in Oman and 28 new fungal species from date fruits. The study discusses the sources of fungi on dates and the presence of potentially mycotoxin producing fungi on date skin and mesocarp.

## Background

Dates palm (*Phoenix dactylifera* L.) is one of the oldest and most important fruit trees in the Middle East [1, 2]. The total worldwide production of dates is around 7.2 million tons, with approximately 5.1 million tons produced by countries in the Middle East [3]. The top 10 producers of dates are Egypt, Iran, Saudi Arabia, Algeria, Iraq, Pakistan, Oman, UAE, Tunisia and Libya. Besides being an important source of vitamins, minerals and other beneficial nutrients, date fruits were the main sources of calories for people living in this part of the world. There are hundreds of date palm cultivars grown in the Middle East, varying in their types from one country to the other. In Oman, there are over 200 different date palm cultivars. *Khalas, Khenizi, Naghal, Burny, Um Al-Sella, Shahla, Mabsali* and *Fardh* are some of the common cultivars in Oman, occupying more than 50% of the area devoted for date palm production [4, 5].

Date fruits are usually harvested and either consumed directly or dried, packed and consumed at a later stage. The fresh and directly consumed dates are referred to as ‘*Rutab*’, while the dried and stored dates are referred to as ‘*Tamar’*. The traditional way of drying dates involves exposing them to direct sun for a certain period of time (few days to weeks). This is followed by packing and storing dates for several months until they are consumed. Since most date palm production in the Middle East is usually within the period from May to October, most people rely on the consumption of fresh dates (*Rutab*) after harvesting. The duration of consumption of fresh dates is variable, as it depends on the cultivars which are grown on a specific location. Some cultivars mature early (e.g. by April to May), while other mature late, sometimes up to October and November. However, after this period, people start consuming the stored dates (*Tamar*) until the next cycle of date’s harvest and production. Some low quality dates are fed to animals because they either come from low quality cultivars or their quality is affected during harvest or storage.

Previous studies reported on the potential contamination of date fruits with some fungal species, including *Aspergillus flavus*, *A. niger*, *Penicillium chrysogenum* and many others [6–10]. These studies raised concerns from the potential contamination of dates with certain mycotoxin-producing fungal species. However, all the previous studies were limited in either being focused on certain fungal types or being dependent on only culture-based approaches for fungal detection [7, 9]. Thus, the amount of information available on the fungal species associated with date fruits is still very limited. This imposes a barrier towards predicting sources of fungal communities and the presence of potentially mycotoxin producing species.

The detection of fungal species in plant material, including date fruits, depended largely on the use of serial dilution or different baiting techniques [7, 8, 10]. However, with the development in molecular techniques, several DNA-based approaches were developed which enabled the detection of several fungal species that are either difficult to grow on synthetic media, or those which are slow growing and usually outgrown by fast growing species. These include the use of pyrosequencing or MiSeq sequencing which made the detection and identification of fungal and bacterial species easy, not only from plant and food material but also from environmental samples such as water and soil [1, 11–15].

The main objective of this study was to characterize the main fungal species associated with dates at the *Tamar* stage. Specific objectives include: (1) to investigate the common fungal species in dates using MiSeq sequencing; and (2) to investigate whether different date parts or date cultivars could differ in their fungal community structure. Understanding fungal diversity in date fruits can help establish a database of the common fungi in these fruits and predict the date fruit parts which are more vulnerable for fungal contamination. It will also help find out the presence of potentially mycotoxin-producing fungi in date fruits.

## Methods

### Collection of samples

The experiment focused on two common date cultivars: *Burny* and *Khenizi. Burny* and *Khenizi* cultivars were grown in Oman in two separate fields, in Ibra and Samail, respectively. Date samples were harvested and immediately exposed to direct sun for approx. 2 weeks. Drying was on the surface of a mat made from dry date leaves. The drying place did not follow any standard hygienic procedures as dates were exposed to natural air without sterilization, which is a usual practice in several places in the Arabian Peninsula. Three different date samples (500 g each) were collected at the *Tamar* stage from each cultivar after partial drying under the sun. The date samples were healthy without any visual symptoms of any disease. The samples were stored in sterile polyethylene plastic bags at 25–30 °C for 3 months prior to analysis. The water activity was measured for each sample using a water activity meter (Ro-tronic Hygrolab, Switzerland). Water activity was measured at the beginning of the storage time and 3 months later (at the microbial analysis time). Three individual date fruits were selected from each cultivar. The skin and the mesocarp of each fruit were separated using sterile forceps and scalpel.

### DNA extraction

DNA was extracted from three skin samples and three mesocarp samples of each date cultivar using the CTAB method with slight modifications [16]. The skin and mesocarp of each sample were ground separately using liquid nitrogen. Then, 0.1 g of date tissue was mixed with 500 μl of pre-warmed 2x CTAB buffer (2% CTAB, 100 mM Tris pH 8.0, 20 mM EDTA pH 8.0, 1.4 M NaCl, 1% PVP-40, 0.2% ß-mercapto-ethanol) and incubated at 65 °C for 30 min. Then 750 μl of phenol: chloroform: isoamyl alcohol (25:24:1) was added to the mixture, vortexed and centrifuged at 10,000 RCF for 15 min. Pre-cooled isopropanol was added to the supernatant and incubated at −40 °C for two hr. Then, the mixture was centrifuged at 10,000 RCF for 5 min and the pellet was washed using 70% ethanol. The DNA pellet was resuspended in 100 μl sterile distilled water and was stored at −60 °C.

### Illumina MiSeq

Illumina MiSeq was carried out for the six samples from each date cultivar. Amplification of samples was carried out in a two-step process, with the first step to amplify genomic regions of interest and the second step to add sequencing adaptors and sample-specific indices to samples. Construction of the forward primer was done using the Illumina i5 sequencing primer (TCGTCGGCAGCGTCAGATGTGTATAAGAGACAG) and the ITS1F primer (CTTGGTCATTTAGAGGAAGTAA) [17]. The reverse primer was constructed with the Illumina i7 sequencing primer (GTCTCGTGGGCTCGGAGATGTGTATAAGAGACAG) and the ITS2aR primer (GCTGCGTTCTTCATCGATGC) [1, 18]. The first PCR was conducted in 25 μl reaction mixture consisting of 1 μl of template DNA, 1 μl of each 5 μM primer and Qiagen HotStar Taq master mix (Qiagen Inc, Valencia, California). The reaction conditions were as follows: an initial denaturation step of 95^○^C for 5 min, then 25 cycles of denaturation at 94^○^C for 30 sec, annealing at 54^○^C for 40 sec, and extension at 72^○^C for 1 min. The final extension was performed at 72^○^C for 10 min.

Products from the first stage amplification were subjected to a second PCR. Primers for the second PCR were designed based on the Illumina Nextera PCR primers as follows: Forward - AATGATACGGCGACCACCGAGATCTACAC[i5index]TCGTCGGCAGCGTC and Reverse - CAAGCAGAAGACGGCATACGAGAT[i7index]GTCTCGTGGGCTCGG. The second stage amplification was run the same as the first stage except for 10 cycles.

Amplification products were visualized and then pooled equimolar. Size selection of each pool was done in two rounds followed by quantification using the Quibit 2.0 fluorometer (Life Technologies). Then it was loaded on an Illumina MiSeq (Illumina, Inc. San Diego, California) 2x300 flow cell at 10pM [19].

### BioInformatic analysis

All sequencing reads were run through Research and Testing Laboratory’s (RTL, Lubbock, TX, USA) standard microbial analysis pipeline. The data analysis pipeline consisted of the denoising and chimera detection stage and the microbial diversity analysis stage. In the first stage, denoising was carried out to remove short sequences, singleton sequences, and noisy reads using the USEARCH [20] and UPARSE [21] algorithms. Then, chimera detection was used to remove chimeric sequences using the UCHIME chimera detection software in *de novo* mode [22]. Finally, the remaining sequences were then corrected per-base to help remove errors in sequencing.

During the diversity analysis stage, all samples were assembled into OTU clusters at 97% identity using the UPARSE [21] algorithm and then globally aligned using the USEARCH [20] global algorithm against a database of high quality ITS fungal gene sequences from GenBank, compiled by RTL, to determine taxonomic classifications. After OTU selection was performed, a phylogenetic tree was constructed in Newick format from a multiple sequence alignment of the OTUs done in MUSCLE [23, 24] and generated in FastTree [25]. Then fungi were classified at the appropriate taxonomic levels using trimmed taxa which takes confidence values into account at each taxonomic level. Individual analysis was carried out for the percentage of sequences assigned to each fungal phylogenetic level for each pooled sample in order to provide the relative abundance for individual samples. The data were filtered at 97% similarity threshold. The mean number of raw reads was 33272, 44517, 40643, 54067 before filtering and 26543, 42272, 37194, 51628 after filtering for *Burny* (mesocarp), *Burny* (skin), *Khenizi* (mesocarp) and *Khenizi* (skin), respectively.

The data were analyzed using the R software [26]. This included the generation of a rarefaction curve plot of the number of OTUs versus the number of sequences, and estimating Richness and Shannon Diversity indices as explained by Kazeeroni and Al-Sadi [1]. Fungal diversity was also estimated using Bray-Curtis similarities followed by analyzing differences in fungal diversity between groups of samples using ‘Permutational Multivariate Analysis of Variance Using Distance Matrices’ function ADONIS [27–29].

### Statistical analysis

Differences among samples in the mean value of water activity were analyzed using Tukey’s Studentized range test (SAS, SAS Institute Inc., USA).

## Results

### Water activity

The water activity of the date samples significantly decreased from 0.65 to 0.60 for *Burny* and 0.62 to 0.59 *Khenizi* from the first day of storage to 3 months after that (at the day of microbial analysis) (*P* < 0.05).

### Fungal diversity estimates

**Fig. 1:**
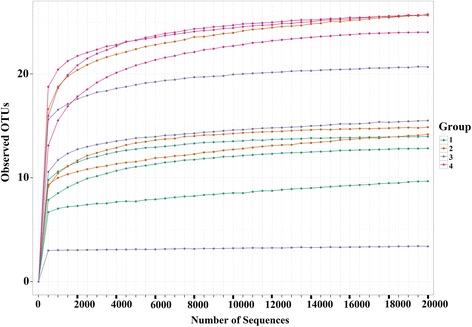
Rarefaction plot of species richness, subsampling from 0 to 20,000 reads in increments of 500 reads. Groups 1, 2, 3 and 4 represent *Burny* (mesocarp), *Burny* (skin), *Khenizi* (mesocarp) and *Khenizi* (skin), respectively

**Fig. 2:**
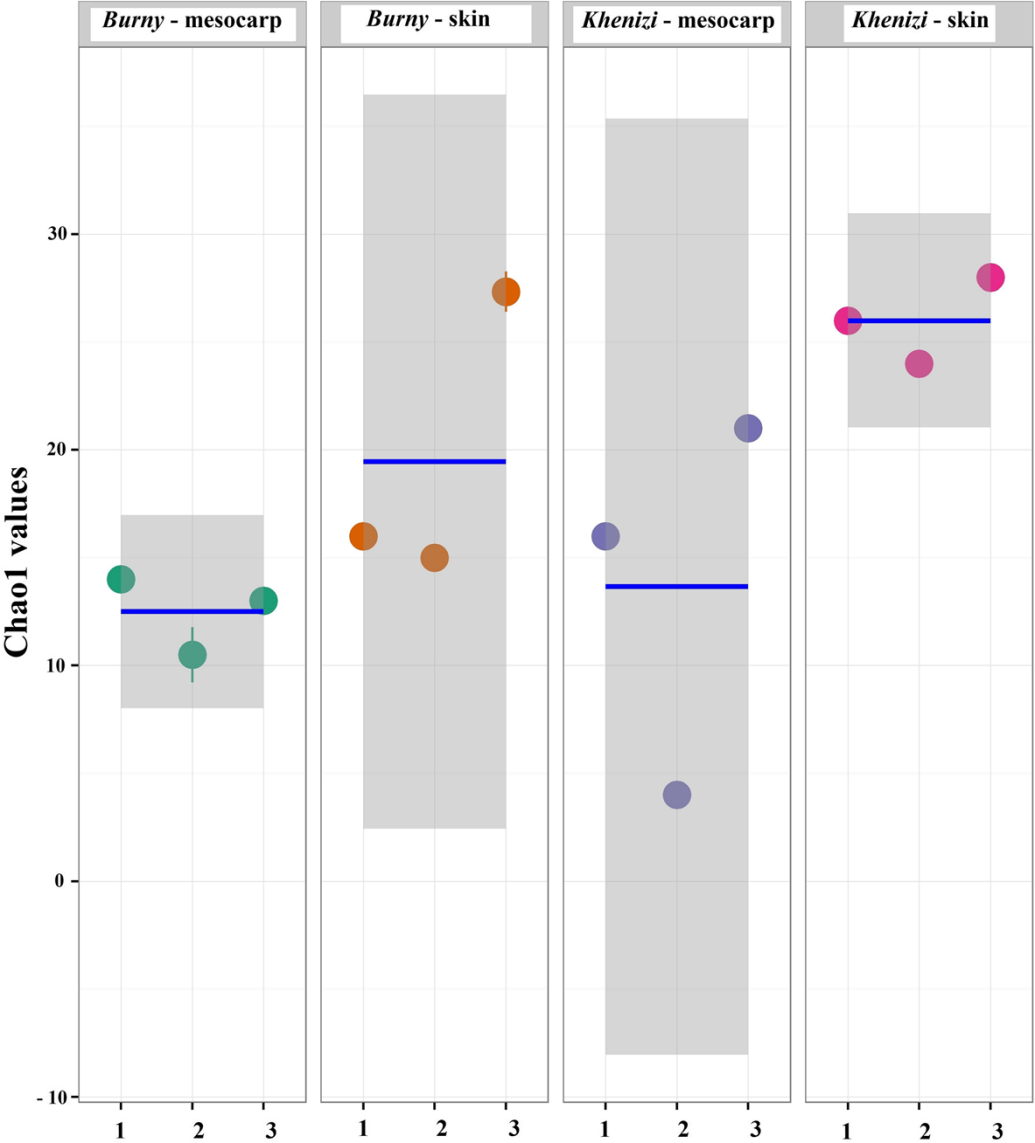
Chao1 richness estimates for the four date samples. The mean value (*line*) and confidence interval (*shaded*) in each group also are illustrated

**Fig. 3:**
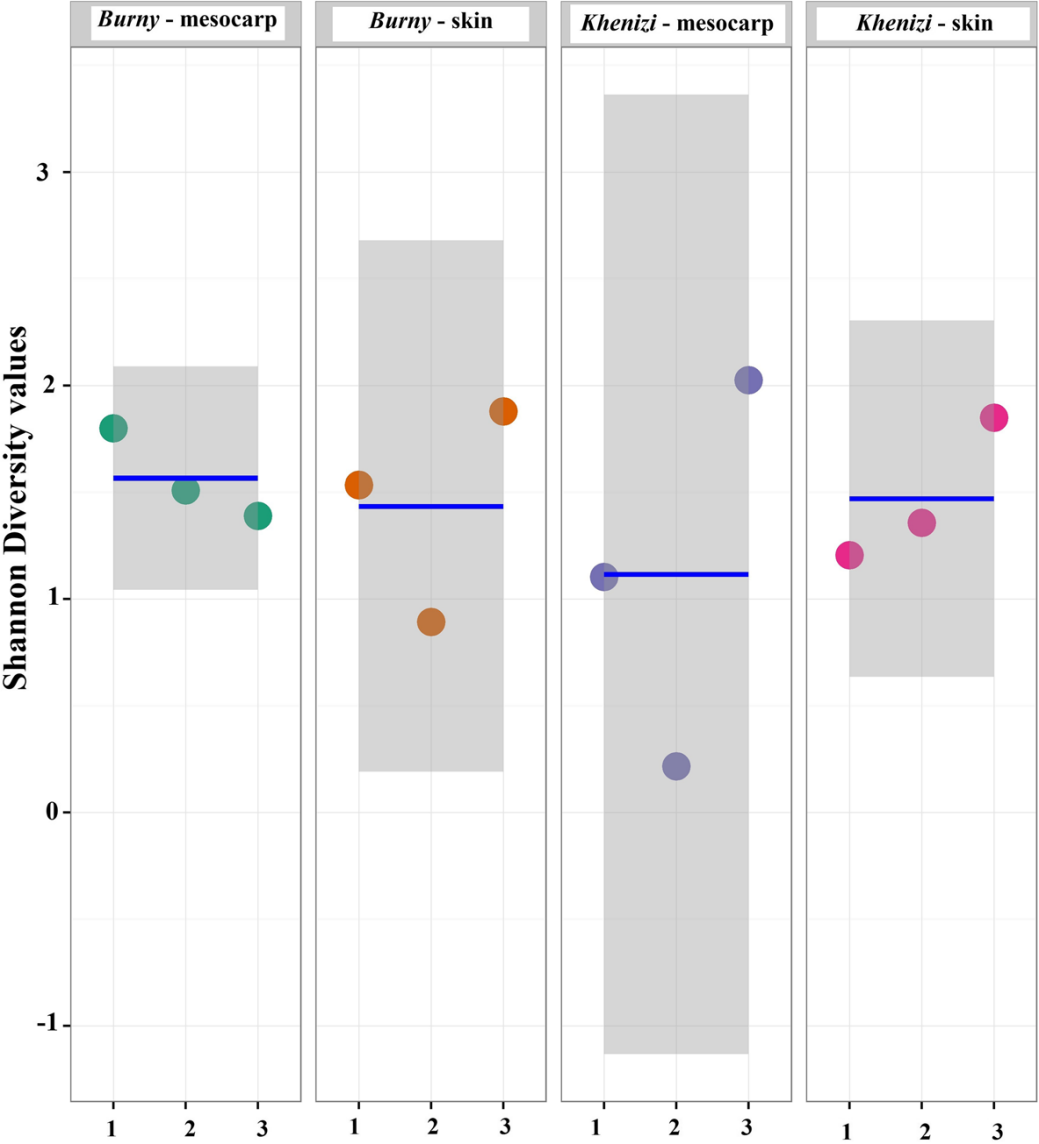
Shannon diversity for the four date samples. The mean value (*line*) and confidence interval (*shaded*) in each group also are illustrated

Analysis showed the presence of variable levels of fungal diversity in the two date cultivars (*Burny* and *Khenizi*) and in the skin and mesocarp of date fruits (Fig. 1). No significant differences were observed in Chao Richness estimates between the mesocarp and skin of date fruits and also between the two cultivars (Fig. 2; *P* = 0.0684), which was due to the slightly high intra-sample diversity within the *Burny*-skin and *Khenizi*-mesocarp treatments. Similarly, no significant differences were observed in Shannon diversity between the fruit cultivars or fruit parts (Fig. 3; *P* = 0.7739).

### Dominant fungal groups

**Fig. 4:**
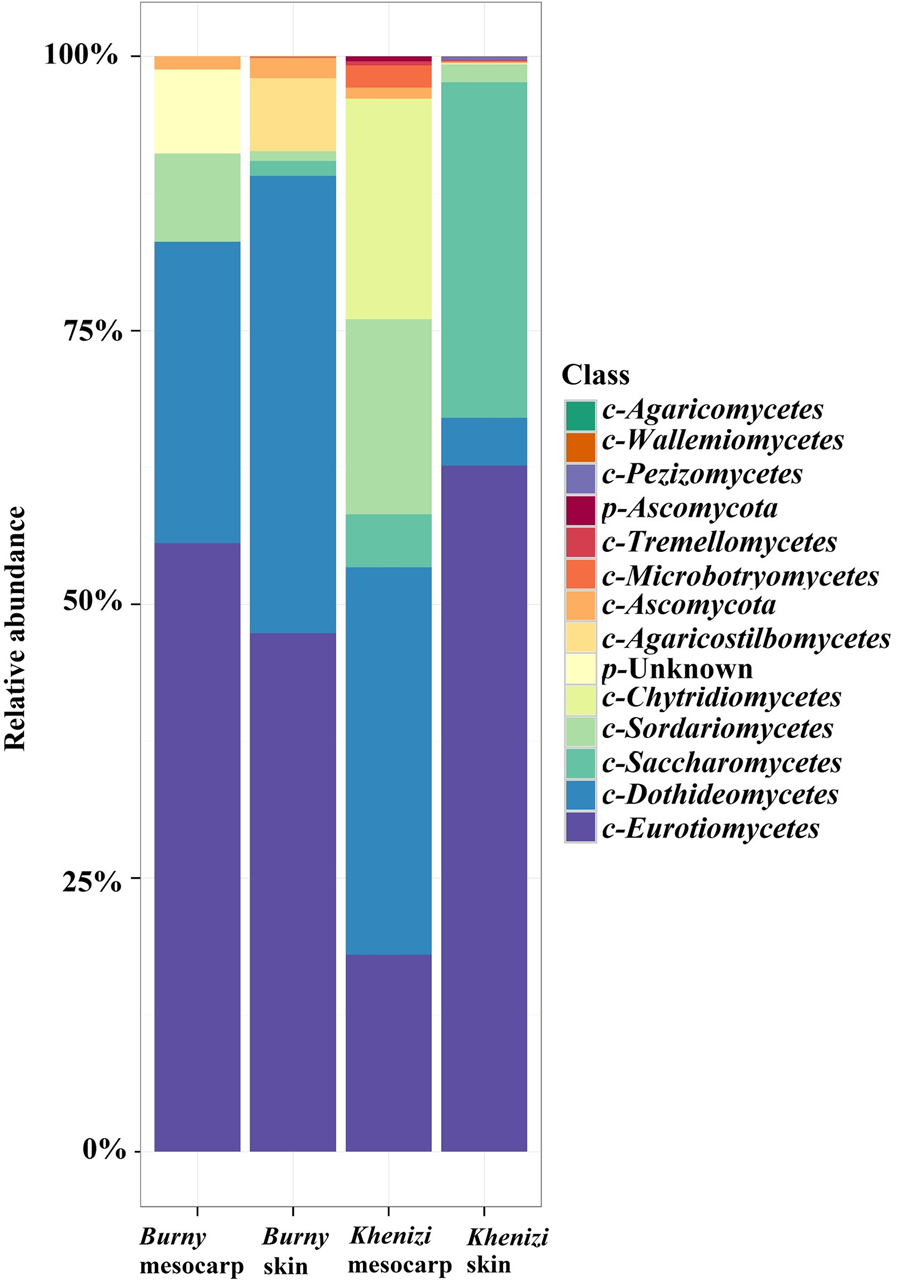
Class-level relative abundance of fungal communities in *Khenizi* and *Burny* dates

*Ascomycota* was the most dominant phylum in the skin and mesocarp of the two date cultivars. It accounted for 81 to over 99% of the fungal reads in the samples. *Basidiomycota* was present in the skin of both cultivars and in the mesocarp of *Khenizi. Chytridiomycota* accounted for 16% of the fungal populations in the mesocarp of *Khenizi* (Fig. 4).

*Eurotiomycetes* was the most dominant fungal class in the samples, followed by *Dothideomycetes*, *Saccharomycetes* and *Sordariomycetes* (Fig. 4). *Eurotiomycetes, Dothideomycetes* and *Sordariomycetes* were detected in all four samples, while the remaining classes were detected in some of the samples. Tremellomycetes was detected in the skin and mesocarp of *Khenizi* but not in *Burny*.

**Fig. 5:**
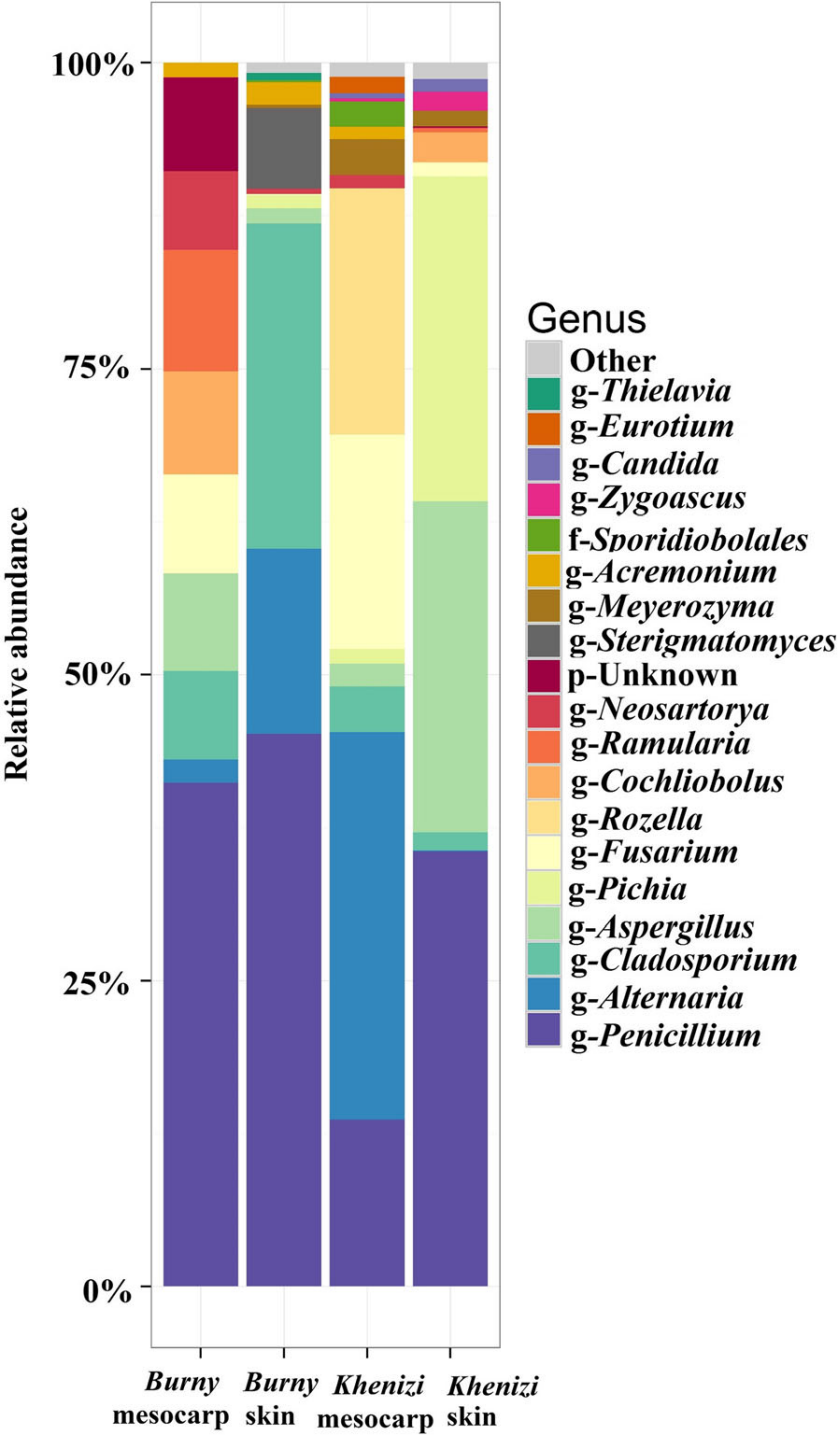
Relative abundance of the 19 most dominant fungal genera in *Khenizi* and *Burny* dates

Analysis of fungal species in the date samples revealed the presence of 54 different fungal species. Eleven of the fungal taxa could not be resolved to the genus or species level, nine were only resolved to the genus level while 34 were identified to the species level (Fig. 5; Table 1). *Penicillium, Alternaria, Cladosporium* and *Aspergillus* species were the most common in most samples. *Penicillium griseofulvum* was the most common fungal species in all samples, making up 13 to 42% of the total fungal reads. This was followed by *Alternaria* sp., *Aspergillus tubingensis*, *Fusarium* sp. and *Cladosporium cladosporioides*.

**Table 1:** Frequency of occurrence of different fungal isolates in the skin and mesocarp of *Burny* and *Khenizi* date cultivars

	*Burny*		*Khenizi*	
Species	Mesocarp	Skin	Mesocarp	Skin
Penicillium griseofulvum*	42.132	40.873	12.511	26.625
*Alternaria* sp	1.698	20.16	40.983	0.168
Aspergillus tubingensis*	7.344	1.105	1.299	37.294
*Fusarium* sp	8.056	0.077	14.909	2.366
*Cladosporium cladosporioides*	3.07	16.768	2.421	1.823
Pichia kudriavzevii*	0	1.44	0.99	16.474
*Rozella* sp	0	0.004	16.612	0
*Cochliobolus* sp	10.004	0.002	0	5.143
*Unknown 1*	3.184	7.221	0.635	0.504
Ramularia eucalypti*	8.891	0	0	0.688
Neosartorya pseudofischeri*	5.848	0.572	0.953	0.129
*Unknown 2*	7.004	0	0	0.048
Sterigmatomyces elviae*	0	5.651	0	0
Meyerozyma guilliermondii*	0	0.181	2.587	2.227
Acremonium implicatum*	1.432	1.58	0.761	0
Cladosporium perangustum*	1.296	1.385	0.209	0.471
Zygoascus meyerae*	0	0.002	0.319	2.243
*Unknown 3*	0	0.134	1.786	0
Candida tropicalis*	0	0	0	1.195
*Eurotium amstelodami*	0	0	1.174	0
*Pichia* sp	0	0	0.014	1.001
Cephaliophora tropica*	0	0	0	0.723
*Unknown 4*	0	0.513	0	0
Exophiala oligosperma*	0	0.432	0.049	0
Penicillium pinophilum*	0	0.438	0	0
Cladosporium sphaerospermum*	0	0.428	0	0
*Alternaria alternata*	0.001	0.4	0.001	0.003
*Aspergillus versicolor*	0	0	0.32	0.065
*Unknown 5*	0	0	0.376	0
*Unknown 6*	0	0	0.356	0
Hannaella sinensis*	0	0	0.335	0
*Aspergillus flavus*	0	0.266	0	0.057
*Unknown 7*	0	0	0	0.221
*Nigrospora* sp	0	0	0	0.192
Myrothecium inundatum*	0	0.177	0	0
*Acremonium* sp	0	0	0.136	0
*Unknown 8*	0	0	0.118	0
Trichoderma asperellum*	0	0	0.109	0.008
Penicillium citrinum*	0.041	0.003	0.032	0.008
*Cladosporium* sp	0	0.076	0	0
Kodamaea ohmeri*	0	0	0	0.073
*Unknown 9*	0	0	0	0.068
Zygosaccharomyces rouxii*	0	0.053	0	0.005
Rhodosporidium kratochvilovae*	0	0	0	0.052
Symbiotaphrina kochii*	0	0	0	0.05
*Unknown 10*	0	0.046	0	0.003
Cryptococcus albidus*	0	0	0	0.036
Candida pimensis*	0	0	0	0.015
*Phoma* sp	0	0.01	0	0
Melanocarpus albomyces*	0	0	0	0.008
Rhodotorula mucilaginosa*	0	0	0	0.008
Wallemia sebi*	0	0	0	0.006
***Penicillium corylophilum***	0	0.004	0	0
*Unknown 11*	0	0	0.002	0

Species in bold are reported in this study for the first time in Oman, while species with (*) symbol are reported for the first time on date fruits. Unknown fungi could not be resolved to the species level. Full data are available through this link http://rtlgenomics.com/ (Project ID: Al-Sadi 4317 Fungal)

Twelve fungal species were detected from the skin and mesocarp of *Burny*, 17 were detected in skin but not the mesocarp and two were detected in mesocarp but not in skin. In *Khenizi*, 17 fungal species occurred in both the skin and mesocarp tissues, 19 occurred only in the skin and 11 occurred only in the mesocarp (Table 1). *Trichoderma asperellum*, *Aspergillus versicolor* and *Pichia* sp were detected only in the mesocarp and skin of *Khenizi* but not in *Burny. Aspergillus flavus* and *Zygosaccharomyces rouxii* were detected only in the skin of *Khenizi* and *Burny* cultivars (Table 1). Some fungal species were detected for the first time in date fruits or in Oman (Table 1).

### Analysis of community composition across samples

PerMANOVA analysis based on Bray-Curtis distances indicated the presence of insignificant differences in the fungal community structure between the mesocarp and skin of *Burny* (*R* ^2^ = 0.346, *P* = 0.150) and *Khenizi* (*R* ^2^ = 0.310, *P* = 0.150) cultivars. Also, no significant differences were observed in the fungal community structure between the *Burny* and *Khenizi* cultivars (Table 2).

**Table 2:** Effect of date parts and date cultivars on fungal diversity revealed using PerMANOVA analysis

Parameter	Treatment	F model	*R* ^2^	*P* adjusted
Date part	Skin X mesocarp (Burny cultivar)	2.113499	0.34571	0.150
	Skin X mesocarp (Khenizi cultivar)	1.799876	0.31033	0.150
Cultivar	Burny X Khenizi (Mesocarp)	1.621053	0.28839	0.150
	Burny X Khenizi (Skin)	2.899156	0.420219	0.150

## Discussion

*Ascomycota* was the most common phylum in the skin and mesocarp of dates. *Ascomycota* is a very common fungal phylum, previously reported to dominate fungal groups in plant tissues and different soil types and fertilizers [1, 7, 11, 13]. Previous studies on date fruits using culture-based techniques also revealed that *Ascomycota* is the dominant phylum in date fruits [6, 8]. Eurotiomycetes was the most dominant class in date fruits, mainly because it contains two of the most dominant genera in date fruits: *Penicillium* and *Aspergillus*.

*Penicillium, Alternaria, Aspergillus* and *Cladosporium* were the most dominant fungal genera in date fruits, comprising more than 60% of the genera observed in date fruits. These fungi, especially *Penicillium* and *Aspergillus*, are very common airborne fungi that produce thousands of spores and they are common on date fruits. Previous studies reported the association of *Alternaria* spp., *Aspergillus* spp., *Cladosporium* spp, *Dreschlera spicifera, Eurotium amstelodami, E. chevalieri, Fusarium* spp., *Mucor racemosus, Myrothecium verrucaria, Penicillium* spp., *Rhizopus stolonifer*, *Ulocladium atrum* and others with date fruits [6–10, 30–32]. In the current study, 28 fungal species appear to be reported for the first time on date fruits, of which 12 were found on skin and mesocarp, 15 were only on skin and one was only in the mesocarp. This indicates that date fruit, especially the outer skin, is exposed to several fungal species.

The majority of the detected fungal taxa in date fruits are either spoilage fungi (e.g. *Alternaria* spp.) or saprophytes (e.g. *Trichoderma asperellum*). Although date palm is known to be affected by several fungal diseases including bayoud disease (*Fusarium oxysporum* f.sp. *albedenis*) and black scorch (*Ceratocystis radicicola*) [4, 33], the causal agents of these diseases were not detected in date fruits. Three potentially mycotoxin producing fungi were detected on date fruits, namely *Aspergillus flavus, A. versicolor* and *Penicillium citrinum*. Although several reports indicated that these are potential mycotoxin-producing fungi in several crops and food types [30, 34–38], our findings showed that they were found to make up less than 0.5% of the total fungal reads in date fruits. In addition, *A. flavus* was only detected in the skin of both cultivars, not in the fleshy part, which may impose less risk on humans. However, more studies should be done in the future to examine the potential presence of mycotoxin and mycotoxin-producing fungi in dates at different stages of maturation and from different cultivars. In addition, it is unclear whether many of the several fungal species detected in this study could impose a potential risk to humans after consuming contaminated dates or they have a possible role in chemical changes in stored date fruits. Future experiments on these fungi could reveal some of their risks or benefits.

Although several fungal species were detected in dates at the *Tamar* stage, no spoilage was observed in any of the date fruits which were subject to analysis. As opposite to dates at the *Rutab* stage which usually spoil quickly because of the high water activity, spoilage of *Tamar* is not common mainly because of the reduced water activity. Findings from this study revealed that water activity in the stored dates decreased for *Burny* and *Khenizi* dates from 0.64 to 0.62 at the storage time to 0.61 and 0.59, respectively 3 months later. Previous studies reported that many of the food spoilage fungi usually grow at water activity ranges from 0.7 to 0.94 [39].

PerMANOVA analysis indicated that fungal communities in the skin of dates are not significantly different from the communities in the mesocarp for both cultivars. This may suggest that fungal species contaminating the outer part of dates’ fruit (skin) may have the ability to grow into the mesocarp. In our study, 39% and 35% of the fungal species contaminating the skin were also detected in the mesocarp of *Burny* and *Khenizi* cultivars, respectively. Contamination of the dates’ skin and mesocarp with the same fungal species could have occurred while dates were on trees or immediately after harvest. This is because drying of dates can reduce water activity to levels that may not favor fungal growth [6–8, 39]. This may impose a problem to consumers, as even if they remove the skin of dates, they may not get rid of all fungi because many of the fungi are in the fleshy part, the mesocarp. It is therefore important to find out the stage at which contamination occurs to help reduce fungal contamination in dates.

Analysis indicated that 23 unique fungal species were observed in *Khenizi* but not in *Burny*, while 7 unique fungal species were observed in *Burny* but not in *Khenizi*. Also, *Penicillium griseofulvum* was found to make up 41–42% of the species in *Burny* compared to 13–27% of the species in *Khenizi*. However, PerMANOVA analyses did not reveal any significant differences in fungal diversity between the two date cultivars (*P* >0.05). Although the dates from the two cultivars were obtained from two different areas, there appears to be no effect of location or cultivars on the fungal community structure of date fruits.

The presence of different fungal species in date fruits as shown by the analyses of alpha diversity (Shannon index, richness estimates) and beta diversity (perMANOVA analysis of Bray-Curtis similarities) raises questions concerning the sources of these fungi. The low level of water activity in dates may lower the chance for dates to be infected at the drying/storage stage. However, the ripening stage of dates is the stage at which contamination by fungi may occur [8, 31]. Since our study did not evaluate this stage, a future study on the possible contamination of dates at different stages of maturation and storage may reveal the stage at which contamination is at high. This may help reduce the chance of date contamination with fungi.

## Conclusion

Alpha-based analyses of fungal diversity in date palm fruits at the *Tamar* stage indicated the presence of different fungal species. The study appears to be the first report of 25 fungal species in Oman and 28 fungal species on date fruits, with some species being potential producers of mycotoxins. Beta analysis of fungal communities showed that they are not related to specific date cultivars or date part (skin and mesocarp), indicating the possible contamination of date cultivars and date parts with the same species of fungi. Future studies should address the source of these fungi in date fruits. They should also address fungal contamination in dates at different stages of maturation/drying and the role of fungi in date spoilage, especially at the *Rutab* stage. In addition, attention should be given to evaluating the effect of date processing on reducing contamination of dates with harmful fungi.

## Abbreviations

CTAB: Cetyltrimethylammonium bromide
ITS: Internal transcribed spacer
OUT: Operational taxonomic unit
